# Auranofin Modulates Thioredoxin Reductase/Nrf2 Signaling in Peripheral Immune Cells and the CNS in a Mouse Model of Relapsing–Remitting EAE

**DOI:** 10.3390/biomedicines11092502

**Published:** 2023-09-10

**Authors:** Layla A. Al-Kharashi, Naif O. Al-Harbi, Sheikh F. Ahmad, Sabry M. Attia, Mohammad M. Algahtani, Khalid E. Ibrahim, Saleh A. Bakheet, Mohammed M. Alanazi, Saleh A. Alqarni, Sary Alsanea, Ahmed Nadeem

**Affiliations:** 1 Department of Pharmacology and Toxicology, College of Pharmacy, King Saud University, Riyadh 11451, Saudi Arabia; 2 Department of Zoology, College of Science, King Saud University, Riyadh 11451, Saudi Arabia

**Keywords:** multiple sclerosis, myeloid immune cells, lymphoid immune cells, auranofin, thioredoxin reductase, Nrf2 signaling

## Abstract

Multiple sclerosis (MS) is one of the most prevalent chronic inflammatory autoimmune diseases. It causes the demyelination of neurons and the subsequent degeneration of the central nervous system (CNS). The infiltration of leukocytes of both myeloid and lymphoid origins from the systemic circulation into the CNS triggers autoimmune reactions through the release of multiple mediators. These mediators include oxidants, pro-inflammatory cytokines, and chemokines which ultimately cause the characteristic plaques observed in MS. Thioredoxin reductase (TrxR) and nuclear factor erythroid 2-related factor 2 (Nrf2) signaling plays a crucial role in the regulation of inflammation by modulating the transcription of antioxidants and the suppression of inflammatory cytokines. The gold compound auranofin (AFN) is known to activate Nrf2 through the inhibition of TrxR; however, the effects of this compound have not been explored in a mouse model of relapsing–remitting MS (RRMS). Therefore, this study explored the influence of AFN on clinical features, TrxR/Nrf2 signaling [heme oxygenase 1 (HO-1), superoxide dismutase 1 (SOD-1)] and oxidative/inflammatory mediators [IL-6, IL-17A, inducible nitric oxide synthase (iNOS), myeloperoxidase (MPO), nitrotyrosine] in peripheral immune cells and the CNS of mice with the RR type of EAE. Our results showed an increase in TrxR activity and a decrease in Nrf2 signaling in SJL/J mice with RR-EAE. The treatment with AFN caused the amelioration of the clinical features of RR-EAE through the elevation of Nrf2 signaling and the subsequent upregulation of the levels of antioxidants as well as the downregulation of oxidative/pro-inflammatory mediators in peripheral immune cells and the CNS. These data suggest that AFN may be beneficial in the treatment of RRMS.

## 1. Introduction

Multiple sclerosis (MS) is a complex disease that is characterized by immune system and CNS dysfunction. This immune-mediated disorder affects millions of people throughout the world, being more prevalent in young adults than in older people [1]. It is one of the costliest inflammatory diseases in the USA as it places a huge burden on the healthcare system that amounts to USD 85 billion/year [2]. MS is characterized by the presence of plaques in the CNS, i.e., in the spinal cord and the brain, that result from the demyelination of neurons due to autoimmune reactions. There are different clinical forms of MS. Relapsing–remitting (RR) MS is the most prevalent form and, if it is not treated on time, may progress to secondary progressive MS. RRMS is characterized by unpredictable demyelinating events that affect the CNS (brain, spinal cord, and optic nerves) causing visual impairment, sensory/coordination issues, cognitive dysfunction, and bowel/bladder incontinence [3,4]. There is an unmet medical need for effective treatments of this disorder, and newer therapeutic approaches need to be developed to improve the clinical symptoms of RRMS.

The cells of the immune system and the CNS have the potential to affect each other. The immune system and the CNS bidirectionally communicate with each other possibly causing blood–brain barrier (BBB) dysfunctions which may further amplify neuroinflammation and the demyelination of neurons [5,6]. Initially, autoimmune reactions involving T cells and other innate cells may participate in the elevation of systemic inflammation which may impair the BBB, leading to the entry of leukocytes into the brain parenchyma. Cells of myeloid/lymphoid origin, e.g., macrophages, DCs, T cells, B cells, and neutrophils, have been implicated in the etiology of MS, as confirmed by several previous studies [7,8,9,10]. These immune cells have the capacity to release several different mediators including reactive oxygen species (ROS), pro-inflammatory cytokines, proteases, and chemokines. All of them together activate microglia and oligodendrocytes in the CNS, which further amplifies the inflammation brought in by systemic immune cells. Cells of the peripheral immune system and the CNS keep activating each other through various feedback loops and cause the progression of the disease. Various immune cells have been shown to be in an activated state both in the peripheral circulation and in the CNS of patients with different forms of MS, i.e., relapsing–remitting (RR) and primary progressive (PP) MS [1,4,7,8,9,11].

Auranofin (3,4,5-triacetyloxy-6-(acetyloxymethyl)oxane-2-thiolate) is a sulphur-containing gold compound which has been utilized against joint inflammation. Auranofin (AFN) is usually considered safe because of its favorable side effect profile and therapeutic effects [12,13]. Auranofin was also tested in multiple inflammatory preclinical disease models and showed great potential to treat a variety of inflammatory immune conditions such as hepatitis, colitis, and Alzheimer’s disease through its antioxidant and anti-inflammatory actions [12,14,15].

AFN has been reported to induce both antioxidant and anti-inflammatory processes through multiple mechanisms, which include TrxR inhibition and Nrf2 activation in peripheral immune cells and the CNS [16]. AFN causes the upregulation of HO-1 and other antioxidant enzymes. On the other hand, AFN also causes the inhibition of inflammation signaling related to the NFkB pathway, such as iNOS- and IL-6-dependent signaling [12,17]. However, its efficacy has not been tested in mouse models of RR-EAE.

PLP_139-151_-induced EAE in SJL/J mice is a classical model for investigating the therapeutic effect of novel compounds. This EAE mouse model shows relapse and remission of clinical symptoms as well as immunological features resembling human RRMS. It is different from MOG_35-55_-induced EAE in C57BL/6 mice, which is a model of secondary progressive MS. PLP_139-151_-induced EAE is a mild disease model, whereas MOG_35-55_-induced EAE is a severe disease model. Cuprizone-induced EAE involves significant demyelination caused by the activation of myeloid immune cells in the CNS, as the involvement of lymphoid immune cells is minimal in this model. Therapies with a potential for inducing remyelination are usually tested in this model [18,19]. SJL/J mice with the RR type of EAE are more suitable for assessing therapeutic effects on disease relapse, whereas C57BL/6 mice with EAE are suitable for the assessment of therapies that have a potential to modify the chronic phase of the disease. However, all these models are needed to investigate the efficacy of novel treatment strategies before their translation in human MS subjects.

There are several key players in the maintenance of the oxidant–antioxidant balance in different immune cells. Nrf2 signaling is one of the key pathways that is involved in the transcription of antioxidant genes when cells are stressed. Nrf2 signaling is controlled by the TrxR1 redox enzyme, which does not allow the translocation of Nrf2 from the cytosol to the nucleus [16,20]. AFN was shown to inhibit TrxR1, thus leading to the nuclear translocation of Nrf2 and the induction of antioxidants such as HO-1 and SOD-2, among hundreds of other important enzymes. Nrf2 signaling is known to control inflammatory pathways related to the induction of neuroinflammation, such as NFkB, iNOS, IL-6, and IL-17A [20,21]. Therefore, AFN may control neuroinflammation through the attenuation of pathways linked to inflammatory cytokines and oxidative enzymes. However, the role of AFN has not been ascertained in murine models of RR-EAE.

Since TrxR1/Nrf2 signaling plays a prominent role in fine tuning the overall antioxidant and anti-inflammatory balance through the control of systemic and neuronal inflammation, which are crucial players in the development of RRMS, the effects of a well-known gold compound, AFN, on the oxidant–antioxidant equilibrium, inflammatory cytokines, and clinical features were tested in an RR-EAE SJL/J mouse model of MS. Our data showed that RR-EAE mice had increased TrxR activity and decreased Nrf2 signaling along with increased levels of cytokines such as IL-6 and IL-17A in peripheral immune cells and the CNS. AFN inhibited TrxR activity, upregulated Nrf2 signaling, and downregulated the levels of inflammatory cytokines, with a concurrent improvement of the clinical features in the RR-EAE mouse model of MS.

## 2. Materials and Methods

### 2.1. Animals

The SJL/J mice utilized in this investigation were obtained from Jackson Laboratories (Bar Harbor, ME, USA). Female mice (9–10 weeks of age) were kept at the Animal Facility of the College of Pharmacy, King Saud University. The animals were housed in a standard sanitary environment, with unrestricted access to food and water, and controlled surroundings (temperature: 24–26 °C; circadian rhythm: 12 light/12 h, light/dark cycle; humidity: 60%) before starting the immunization protocol. The protocols for conducting the experiments were approved by the Institutional Animal Care and Use Committee, King Saud University.

### 2.2. Development of Relapsing–Remitting (RR) Experimental Autoimmune Encephalomyelitis (EAE) in SJL/J Mice

For the experimental development of the RR type of EAE in SJL/J mice, the mice were administered a dose of 200 µg of myelin proteolipid protein 139–151 (PLP_139-151_) peptide emulsified in CFA [Hooke Laboratories, Lawrence, MA, USA]. On the day of the immunization with PLP_139-151_, a dose of 200 ng of pertussis toxin (Hooke Laboratories, Lawrence, MA, USA) was also injected intraperitoneally (i.p.) to each mouse. The assessment of the clinical features of the RR type of EAE was conducted according to the following criteria: 0, no disease symptoms; 1, complete paralysis of the tail; 2, partial hind paralysis/weakness; 3, complete hind limb paralysis; 4, front and hind limb paralysis; and 5, moribund state. If any animal reported a score above 4, it was removed from the study.

### 2.3. Experimental Groups

To assess the role of AFN on systemic and neuronal inflammation in SJL/J mice, the mice were administered AFN at a dose of 5 mg/kg, i.p. (Sigma Chemicals, St. Louis, MO, USA) in a volume of 2.5 mL/kg or the vehicle (5% DMSO in normal saline), five times a week (Sunday to Thursday, once in the afternoon) from day 10 through day 41 post-immunization. After the onset of the clinical symptoms, the SJL/J mice were randomly divided into one of the following cohorts: **Cohort 1:** vehicle-administered control group (Veh), i.e., non-immunized mice that were administered only the drug vehicle; **Cohort 2:** auranofin-administered control mice (AFN), i.e., non-immunized mice that were administered AFN at a dose of 5 mg/kg, i.p., as indicated above; **Cohort 3:** vehicle-administered diseased mice (EAE), i.e., PLP-immunized mice that were administered only the drug vehicle as indicated above; **Cohort 4:** auranofin-administered diseased mice (AFN + EAE), i.e., PLP-immunized mice that were administered AFN at a dose of 5 mg/kg, i.p., as indicated above. The mice were sacrificed by isoflurane inhalational anesthesia on day 42, and the brain/spinal cord/spleen were isolated for various molecular/biochemical analyses, as detailed below.

### 2.4. Real-Time PCR

The cortex was isolated in RNAlater and kept at -20 °C for a month before being used for the isolation of RNA. Total RNA (1 μg) was converted in cDNA through reverse transcription (High-Capacity cDNA archive kit, Applied Biosystems, Grand Island, NY, USA) according to the manufacturer’s protocol [22]. The mRNA expression of HO-1 (Forward: GAGCAGAACCAGCCTGAACT; Reverse: GCCTTCTCTGGACACCTGAC), SOD-1 (Forward: TGGGGACAATACACAAGGCTGT; Reverse: TTTCCACCTTTGCCCAAGTCA), Nrf2 (Forward: TGCTCATTGTGGTAGGCAGG; Reverse: GGGAAAGGCACAGAGAGCAT), IL-6 (Forward: TCCAGTTGCCTTCTTGGGAC; Reverse: AGTCTCCTCTCCGGACTTGT), iNOS (Forward: CTATGGCCGCTTTGATGTGC); Reverse: CAACCTTGGTGTTGAAGGCG), and GAPDH (Forward: GGCAAATTCAACGGCACAGT; Reverse: TGAAGTCGCAGGAGACAACC) was evaluated by real-time PCR on an ABI PRISM 7500 sequence detection system (Applied Biosystems, Grand Island, NY, USA) using GenScript primers (Piscataway, NJ, USA) [22]. All primer sequences are written in the 5′-3′ direction. The fold difference in gene expression for the different groups was measured by the delta-delta Ct method [23].

### 2.5. Protein Expression Analsyis by Flow Cytometry in Splenocytes

The spleens were extracted on the day of sacrifice from the different groups of mice to prepare single-cell suspension. Briefly, the spleens were cut into large pieces and then crushed with a 2 mL syringe plunger through a 100 µm stainless steel sieve, followed by the removal of clumps with a syringe for multiple times, ultimately sieving the samples through a 70 µm stainless strainer to obtain single-cell suspensions in RPMI-1640, as reported earlier [22,24]. The RBCs in the suspension were lysed, and then the leukocytes were immunostained with monoclonal antibodies against cell surface markers such as CD3 (clone: 17A2; APC-Cy7/FITC; BioLegend, San Diego, CA, USA) or CD11b (Clone M1/70; APC/FITC; BioLegend, San Diego, CA, USA). After standard fixation and permeabilization, the leukocytes (Miltenyi Biotech, Bergisch Gladbach, North Rhine-Westphalia, Germany) were immunostained with fluorophore-linked monoclonal/polyclonal antibodies against intracellular proteins such as TrxR1 (clone: B2; Alexa Fluor^®^ 647; Santacruz Biotech, Dallas, TX, USA), IL-17A (Clone: TC11-18H10.1; PE-Dazzle; BioLegend, San Diego, CA, USA), IL-6 (Clone: MP5-20F3; PE; BioLegend, San Diego, CA, USA), iNOS (clone: C11; Alexa Fluor^®^ 488; Santacruz Biotech, Dallas, TX, USA), Nrf2 (clone: D1Z9C; PE; Cell Signaling Tech, Danvers, MA, USA), HO-1 (clone: F4; Alexa Fluor^®^ 647; Santacruz Biotech, Dallas, TX, USA), 3-nitrotyrosine (Clone: 39B6; Alexa Fluor^®^ 647; Santacruz Biotech, Dallas, TX, USA), USA). CD11b was used as a general marker for myeloid immune cells, as it is expressed on neutrophils, DCs, monocytes, and macrophages [25]. The immunolabeled leukocytes obtained from the spleen were then analyzed on a flow cytometer (Beckman Coulter, Brea, CA, USA) for cell surface and intracellular proteins, according to the characteristics of the antibody-coupled fluorophores, using Cytomics FC 500 software, as described before [22,24,26].

### 2.6. Evaluation of TrxR Activity in the CNS

TrxR activity was assessed in the cerebral cortex on the basis of the DTNB/NADPH redox cycle reactions. Briefly, reaction mixtures (200 μL) of the samples, NADPH, and DTNB in 100 mM/1 mM potassium phosphate/EDTA buffer (pH-7) were loaded onto a 96-well plate. TrxR present in the samples catalyzed the reduction of DTNB to 5-thio-2-nitrobenzoic acid (TNB) using NADPH, whose absorbance was measured at 412 nm using a microplate reader. The TrxR activity in each sample was normalized according to its protein content. The results are expressed as nmol NAPDH oxidized/min/mg protein.

### 2.7. Evaluation of Nrf2 Binding Activity in the CNS by ELISA

The measurement of Nrf2 binding to its antioxidant response element (ARE) in the cerebral cortex was determined using an TransAM ELISA kit (Active Motif, Carlsbad, CA, USA), according to the manufacturer’s instructions.

### 2.8. Evaluation of p-NFkB, Lipid Peroxides, and Myeloperoxidase Activity in the CNS

The measurement of phosphorylated NF*k*B (Pathscan^®^ Phospho-NFkB) in the spinal cord was performed using an ELISA kit (Cell Signaling Technology, Danvers, MA, USA), according to the manufacturer’s instructions. MPO activity in the spinal cord was evaluated as an indicator of neutrophilic inflammation, as stated earlier [22,24,26]. The levels of lipid peroxides were measured in the spinal cord as described earlier [22].

### 2.9. Statistical Analysis

The results are expressed as mean± SEM. Comparisons among different groups (Veh, AFN, EAE and AFN + EAE) for the measured parameters were carried out by ANOVA (analysis of variance) followed by Tukey’s multiple comparison tests. The data in this study were derived from two independent experiments. The area under the curve (AUC) was also analyzed for the measurement of overall disease severity in the EAE and AFN + EAE groups, and these two groups were compared by unpaired *t*-test. The results were considered statistically significant if *p* < 0.05. All statistical analyses were conducted using Graphpad Prism 9 (San Diego, CA, USA).

## 3. Results

### 3.1. AFN Leads to the Amelioration of the Clinical Symptoms in SJL/J Mice with the RR Type of EAE 

**Figure 1:**
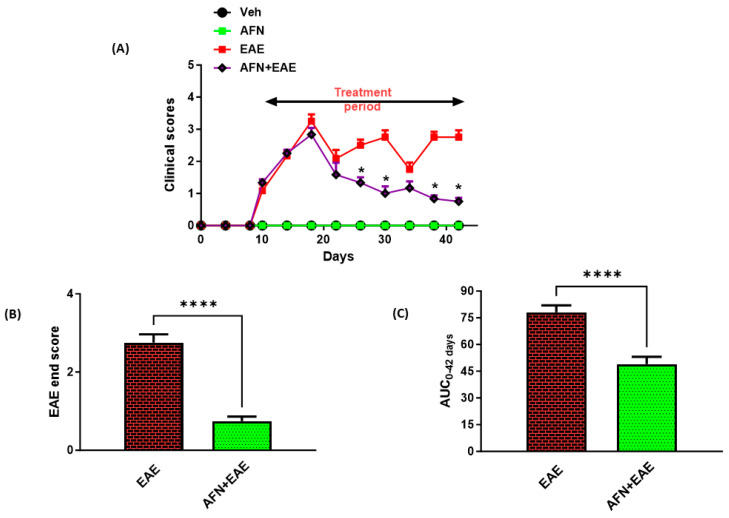
Treatment with AFN improves the clinical features in an RR model of EAE in SJL/J mice. (**A**) Clinical symptoms recorded during the experiment, (**B**) AUC from day 0 to day 42, and (**C**) clinical score at the end of the study. Control (Veh) and diseased (EAE) mice were treated with auranofin at 5 mg/kg, i.p. (5 times/week) for a month, and the clinical symptoms were recorded in all groups during the month-long treatment. Data are expressed as mean ± SEM, n = 6–8. * *p* < 0.05 vs. EAE group; **** *p* < 0.0001.

AFN is a gold-containing compound which has been tested in different inflammatory animal models due to its potent antioxidant and anti-inflammatory actions. Therefore, we tested its efficacy in an RR model of EAE in SJL/J mice. Our data showed that AFN, which was administered for about a month, started showing its efficacy after 10 days from its administration. AFN affected classical features of RR-EAE by attenuating the first relapse and showed efficacy until the end of the treatment (Figure 1A). There was a significant difference in the end scores between AFN-treated EAE mice and vehicle-treated EAE mice. Further, the AUC from day 0 to day 42 between AFN-treated EAE mice and vehicle-treated EAE mice was significantly different, indicating the overall inhibition of the disease symptoms by AFN (Figure 1B,C). These observations suggested that AFN attenuated the clinical features in the examined RR model of EAE in SJL/J mice.

### 3.2. AFN Causes the Inhibition of TrxR Activity and the Upregulation of Nrf2 in the CNS of Immunized SJL/J Mice

**Figure 2:**
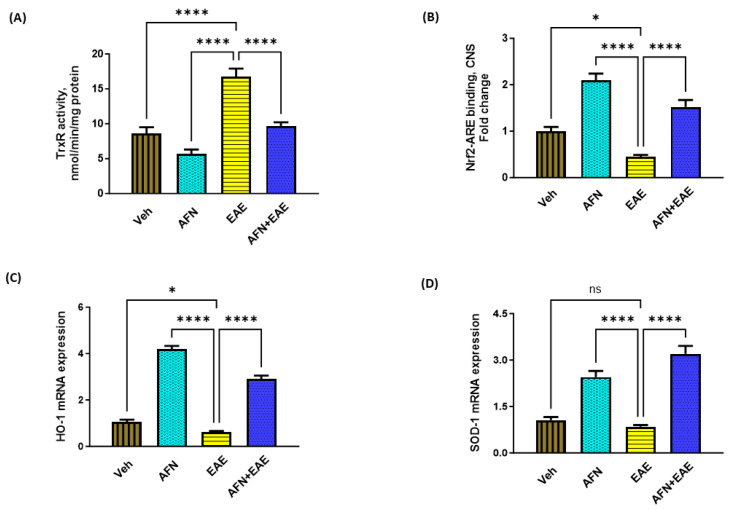
Treatment with AFN causes an elevation in Nrf2 signaling in the CNS of EAE mice. (**A**) TrxR activity, (**B**) Nrf2–ARE binding activity, (**C**) HO-1 mRNA levels, and (**D**) SOD-1 mRNA levels. Control (Veh) and diseased (EAE) mice were treated with auranofin at 5 mg/kg, i.p. (5 times/week) for a month, and biochemical/molecular assessments in the CNS were carried out in all groups at the end of the study. Data are expressed as mean ± SEM, n = 6. * *p* < 0.05; **** *p* < 0.0001; ns = not significant.

It was reported that AFN exerted antioxidant/anti-inflammatory actions through the inhibition of TrxR activity; therefore, we first sought to determine the effect of AFN on TrxR activity in the brain. Our data showed that TrxR activity was significantly elevated in mice with EAE as compared to vehicle-treated control mice (Figure 2A); however, the treatment with AFN led to the inhibition of TrxR activity, indicating that AFN was able to cross the BBB and reach the CNS. Next, we analyzed Nrf2 signaling in the brain, as it is thought to be under the regulation of TrxR. Our data showed that Nrf2 binding to its ARE as well as its signaling were significantly increased by AFN in mice with EAE (Figure 2B). Nrf2-related antioxidant genes such as HO-1 and SOD-2 were also upregulated by AFN in mice with EAE (Figure 2B,C). These data showed that AFN has the potential to activate Nrf2 signaling in the CNS of EAE mice through TrxR inhibition.

### 3.3. AFN Causes the Inhibition of NFkB Signaling in the CNS of Immunized SJL/J Mice

**Figure 3:**
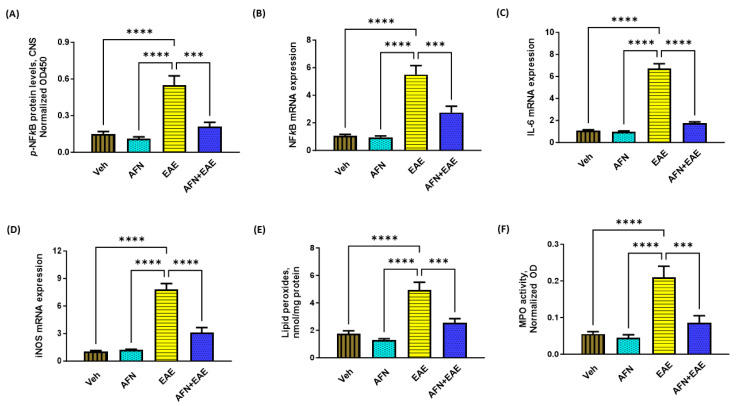
Treatment with AFN causes a reduction in oxidative mediators in the CNS of EAE mice. (**A**) p-NFkB protein levels, (**B**) NFkB mRNA levels, (**C**) IL-6 mRNA expression, (**D**) iNOS mRNA levels, (**E**) lipid peroxides levels, and (**F**) MPO activity. Control (Veh) and diseased (EAE) mice were treated with auranofin at 5 mg/kg, i.p. (5 times/week) for a month, and biochemical/molecular assessments in the CNS were carried out in all groups at the end of the study. Data are expressed as mean ± SEM, n = 6. *** *p* < 0.001; **** *p* < 0.0001.

As Nrf2 signaling is known to suppress inflammatory mediators, we first measured the levels of *p*-NFkB in the CNS of all groups. Our data showed that the *p-*NFkB protein and the NF*k*B mRNA levels were markedly increased in mice with EAE as compared to non-diseased control mice (Figure 3A,B). Treatment with AFN caused a significant downregulation of the *p*-NFkB levels in the CNS of mice with EAE. Further, the levels of mediators associated with NF*k*B signaling such as iNOS and IL-6 were also markedly attenuated by AFN treatment in mice with EAE (Figure 3C,D). Oxidative stress markers such as lipid peroxides and MPO activity were also significantly reduced by AFN treatment in mice with EAE (Figure 3E,F). These observations suggested that AFN has the potential to suppress NFkB -related oxidative/inflammatory in the CNS of EAE mice.

### 3.4. AFN Causes the Upregulation of Nrf2 Signaling in Peripheral Myeloid Immune Cells in Immunized SJL/J Mice

**Figure 4:**
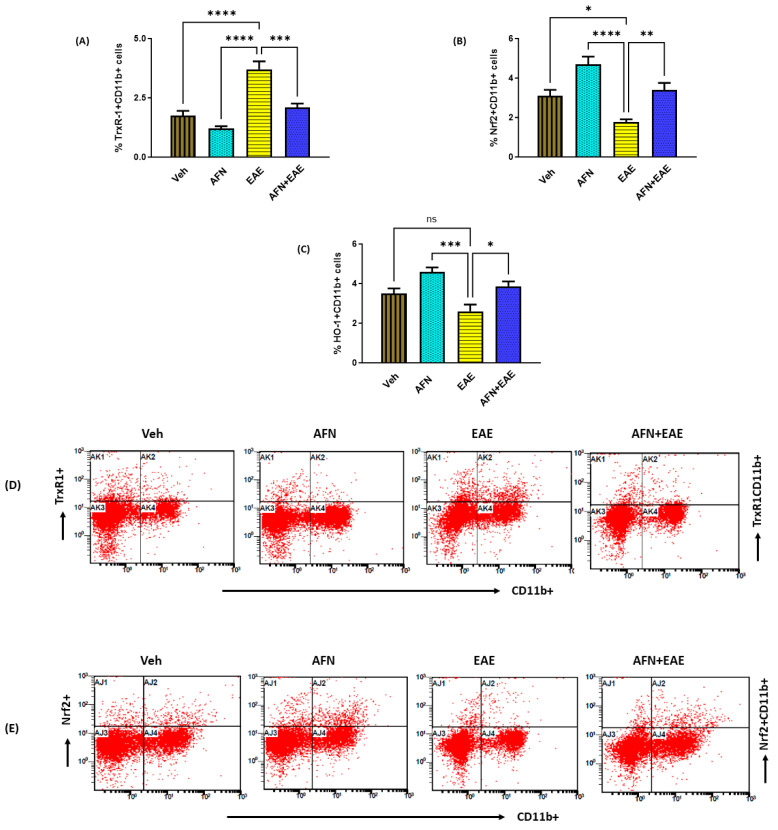
Treatment with AFN causes an elevation in Nrf2 signaling in peripheral myeloid immune cells in EAE mice. (**A**) % of TrxR1 + CD11b+ cells, (**B**) % of Nrf2+ CD11b+ cells, (**C**) % of HO-1+ CD11b+ cells, (**D**) an illustrative flow plot displaying the immunostaining of TrxR1 + CD11b+ cells, and (**E**) an illustrative flow plot displaying the immunostaining of Nrf2 + CD11b+ cells. Control (Veh) and diseased (EAE) mice were treated with auranofin at 5 mg/kg, i.p. (5 times/week) for a month, and biochemical/molecular assessments in the spleen were carried out in all groups at the end of the study. Data are expressed as mean ± SEM, n = 6. * *p* < 0.05; ** *p* < 0.01; *** *p* < 0.001; **** *p* < 0.0001; ns = not significant.

As the immune system plays a significant role in MS initiation and progression, we next sought to determine the effects of AFN on peripheral immune cells of myeloid origin. Our data showed that the AFN treatment led to the inhibition of TrxR1 expression in myeloid (CD11b+) immune cells, as reflected by the decreased % of TrxR1 + CD11b+ cells in mice with RR-EAE (Figure 4A). Our data showed that the AFN treatment led to the activation of Nrf2 signaling in myeloid (CD11b+) immune cells, as reflected by the increased % of Nrf2 + CD11b+ cells in mice with RR-EAE (Figure 4B). Further, the activation of Nrf2 signaling by AFN in the EAE group was associated with antioxidant upregulation, as depicted by the increased HO-1 expression in CD11b+ cells (Figure 4C). These data showed that AFN caused the upregulation of Nrf2 signaling in peripheral immune cells to counteract the oxidative inflammation observed during RR-EAE.

### 3.5. AFN Causes the Downregulation of Oxidative Stress and Inflammatory Mediators in Peripheral Myeloid Cells in Immunized SJL/J Mice

**Figure 5:**
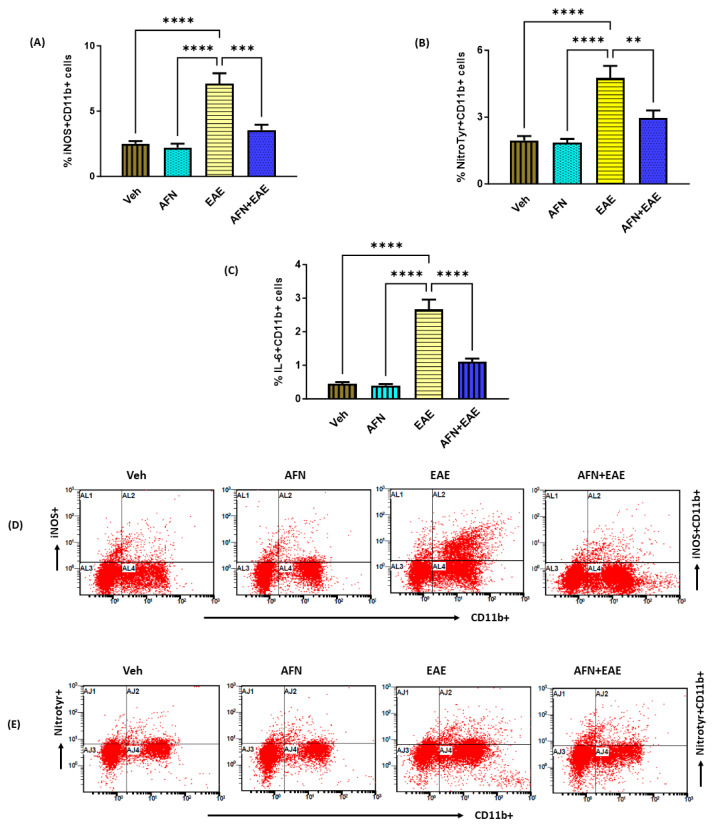
Treatment with AFN causes a reduction in oxidative and inflammatory mediators in peripheral myeloid immune cells in EAE mice. (**A**) % of iNOS + CD11b+ cells, (**B**) % of nitrotyr+ CD11b+ cells, (**C**) % of IL-6+ CD11b+, (**D**) an illustrative flow plot displaying the immunostaining of iNOS + CD11b+ cells, and (**E**) an illustrative flow plot displaying the immunostaining of nitrotyr + CD11b+ cells. Control (Veh) and diseased (EAE) mice were treated with auranofin at 5 mg/kg, i.p. (5 times/week) for a month, and biochemical/molecular assessments in the spleen were carried out in all groups at the end of the study. Data are expressed as mean ± SEM, n = 6. ** *p* < 0.01; *** *p* < 0.001; **** *p* < 0.0001.

We further verified whether AFN had the potential to suppress oxidative stress and inflammatory mediators in peripheral myeloid immune cells. Our data showed that there was an increase in inflammatory and oxidative mediators in myeloid immune cells, as displayed by the increased % of IL-6+, iNOS+, and nitrotyrosine+ CD11b+ myeloid immune cells (Figure 5A–C). Further, the AFN treatment led to the inhibition of both inflammatory and oxidative inflammatory markers, as depicted by the decreased % of IL-6+, iNOS+, and nitrotyrosine+ CD11b+ cells in mice with RR-EAE (Figure 5A–C). These data showed that AFN caused a downregulation of peripheral oxidative stress and inflammation in myeloid immune cells in mice with RR-EAE.

### 3.6. AFN Causes the Upregulation of Nrf2 Signaling in Peripheral Lymphoid Immune Cells (CD3+ T Cells) in Immunized SJL/J Mice

**Figure 6:**
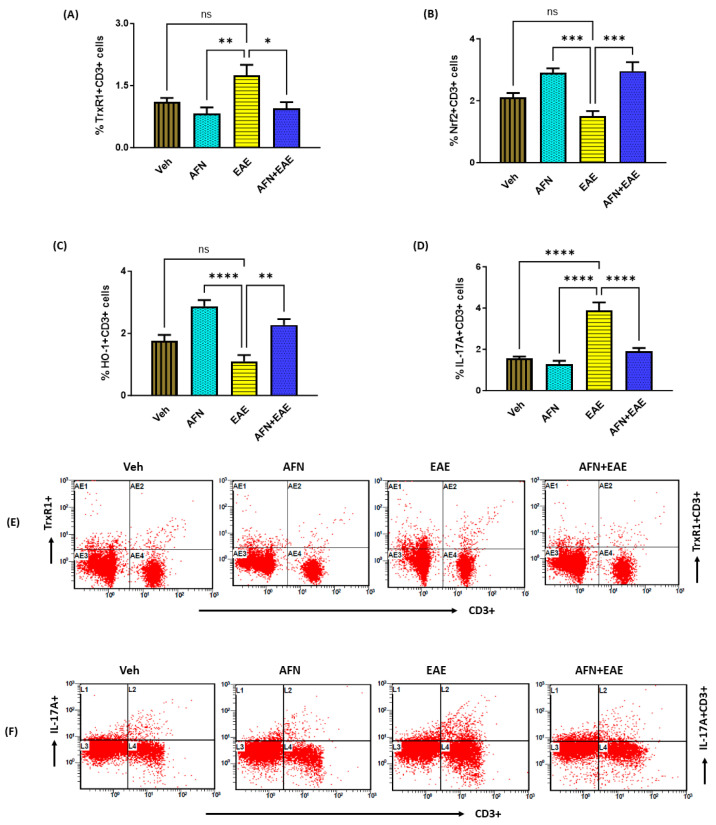
Treatment with AFN elevates Nrf2 signaling and reduces inflammatory mediators in peripheral T cells in EAE mice. (**A**) % of TrxR1 + CD3+ T cells, (**B**) % of Nrf2 + CD3+ T cells, (**C**) % of HO-1 + CD3+ T cells, (**D**) % of IL-17 + CD3+ T cells, (**E**) an illustrative flow plot displaying the immunostaining of TrxR1+ and CD3+ cells, and (**F**) an illustrative flow plot displaying the immunostaining of IL-17A+ and CD3+ cells. Control (Veh) and diseased (EAE) mice were treated with auranofin at 5 mg/kg, i.p. (5 times/week) for a month, and biochemical/molecular assessments in the spleen were carried out in all groups at the end of the study. Data are expressed as mean ± SEM, n = 6. * *p* < 0.05; ** *p* < 0.01; *** *p* < 0.001; **** *p* < 0.0001; ns = not significant.

As T cells also play an important function in the development of EAE in the examined mouse model, we next sought to determine the effects of AFN on peripheral immune cells of lymphoid origin, i.e., T cells. Our data showed that the AFN treatment led to the inhibition of TrxR1 expression in lymphoid immune cells (CD3+), as reflected by the decreased % of TrxR1 + CD3+ T cells in mice with RR-EAE (Figure 6A). Further, the reduction in TrxR1 caused by AFN led to the activation of Nrf2 signaling in T cells, as reflected by the increased % of Nrf2 + CD3+ and HO-1 + CD3+ cells in mice with RR-EAE (Figure 6B,C). Furthermore, the activation of Nrf2 signaling by AFN in the EAE group also caused the downregulation of IL-17A in T cells, as displayed by the decreased % of IL-17A + CD3+ T cells (Figure 6D). These data showed that the AFN-mediated downregulation of IL-17A in T cells could possibly be due to the upregulation of Nrf2 signaling in mice with RR-EAE.

## 4. Discussion

TrxR is a very crucial antioxidant enzyme that is critical in the modulation of various redox-sensitive molecules, including Nrf2, in various immune cells such as T cells, neutrophils, and DCs. A dysfunction in TrxR has been linked to many different inflammatory diseases including neurological disorders [16,22,27]. Our study showed increased TrxR1 expression and TrxR activity with a concomitant reduction in Nrf2 signaling in the periphery and CNS, respectively, in an RR model of EAE. AFN treatment caused a reduction in TrxR activity that caused the activation of Nrf2 signaling in both peripheral immune cells and the CNS of EAE mice. This is the first study showing the effect of AFN in a mouse model of EAE. AFN also induced the upregulation of Nrf2-related signaling in normal mice through the downregulation of TrxR activity. Therefore, it is possible that AFN exerted its effects through TrxR/Nrf2 signaling in both control and EAE mice.

Oxidants are generated during normal metabolism as well as by specialized oxidative enzymes during inflammatory events. In normal healthy conditions, oxidants are scavenged by different antioxidants, ubiquitously present within the immune cells [4,28]. However, when the immune cells are activated, they generate elevated levels of oxidants such as superoxide, nitric oxide, and hydrogen peroxide, which produce other secondary oxidants such as peroxynitrite and hypochlorous acid, due to the presence of iNOS, NOX, and MPO in macrophages, DCs, and neutrophils. Microglial cells also generate increased oxidant levels upon activation by different stimuli. The CNS is specifically susceptible to oxidants generated by infiltrated immune cells and resident microglial due to its high lipid content, ultimately undergoing oxidative damage, which could be responsible for the axonal loss and demyelination observed in RRMS [29]. MS patients are also reported to have upregulated levels of oxidative stress in the peripheral circulation and the brain [4,29,30]. Similarly, mice with EAE displayed increased oxidative stress in different immune cells [22,31]. Our study showed increased levels of markers of oxidative stress in peripheral immune cells and the CNS, which was considerably attenuated by AFN treatment. This could be due to AFN-mediated activation of Nrf2 signaling in the periphery and CNS, which could cause the attenuation of oxidative stress.

Cells of myeloid origin that include DCs, macrophages, and neutrophils contribute significantly to the initiation and progression of MS [32,33,34,35]. Nrf2 signaling is very active in different myeloid cells due to their increased oxidative potential in inflammatory situations. Nrf2 signaling is a master controller of the redox status of a cell and is switched on during conditions of oxidative stress, thereby causing the upregulation of antioxidant genes [36,37]. Our study showed an elevation in the levels of enzymatic antioxidants such as HO-1 and SOD in both peripheral immune cells and the CNS induced by AFN treatment in the examined RR model of EAE, which could be responsible for the suppression of oxidative stress and the improvement of the clinical symptoms. Recent studies also reported an amelioration of the clinical symptoms due to the activation of activation of Nrf2 signaling in EAE models [22,31].

A recent study showed that AFN did not improve the clinical symptoms in an EAE model when it was administered on day 10 post immunization [38]. The differences between the study by Yu et al. [38] and the present study could be due to multiple reasons. Firstly, the previous study used MOG-induced EAE, which is a severe model of EAE, whereas this study utilized PLP-induced EAE, which is a mild form of EAE. Furthermore, Yu et al. [38] used AFN at lower doses (up to 400 µg/kg) compared to us, and it is likely that these low doses of AFN administered therapeutically (starting day 10) were unable to ameliorate the clinical symptoms in their model of severe EAE. In contrast, this study administered a higher dose of AFN (5 mg/kg), which was able to control the milder form of EAE in our model. Furthermore, the previous study ended on day 25 day, whereas our study was extended up to 42 days. All these factors could contribute to the differences between this study and the previous study by Yu et al. [38]. However, further studies using both models and different dosage regimens are required to reach a conclusion.

Lymphoid immune cells such as T cell also play a critical role in the development of the autoimmune inflammation observed in MS. IL-6 is required for the polarization of Th0 (naïve CD4+ T cells) into Th17 cells, which express and release several inflammatory cytokines including IL-17A [39,40]. Antigen-presenting cells such as macrophages and DCs are known to secrete IL-6 during autoimmune reactions, which along with costimulatory signals may be responsible for Th17 cell differentiation, as observed in our study. Past studies showed the involvement of Th17 cells in the etiology of MS in humans and of EAE in animals [39,41,42,43]. AFN was reported to cause a reduction in IL-6 levels; therefore, the reduction in IL-17A expression in CD4+ T cells could be due to a reduction in IL-6 from peripheral myeloid cells. This could result from the upregulation of Nrf2 signaling in T cells, which was shown to reduce Th17-related immune responses in different preclinical disease models including an EAE model [22,24,26,38,44,45].

Neutrophils are the most abundant myeloid cells in the periphery, which makes them crucial players in neuroinflammatory processes through sophisticated crosstalk [46]. This crosstalk could occur between neutrophils and other immune cells, thereby affecting the function and homeostasis of macrophages, T/B cell, and platelets [10,47]. Neutrophils are primed/activated by multiple stimuli (e.g., chemokines, cytokines) due to their expression of different types of receptors such as TLRs, c-type lectin receptors, complement receptors, and G protein-coupled receptors [11,34,43,46]. Neutrophils carry out various effector functions such as phagocytosis, degranulation, oxidative burst, neutrophil extracellular traps (NET) formation, and chemotaxis [10,32]. iNOS and MPO contained in neutrophils may be destructive during the autoimmune inflammation observed in RR-EAE. This might lead to the dysfunction of endothelial cells adjacent to neutrophils due to the elevated levels of peroxynitrite that results in an increased expression of adhesion molecules [22,32,44,45,48]. Such cells might represent a preferential site for adhesion and migration of additional neutrophils as well as for transendothelial migration, which could be responsible for BBB impairment [48,49]. Our study showed that the levels of 3-nitrotyrosine and iNOS were attenuated by AFN in peripheral neutrophils in mice with RR-EAE. This suggested that the oxidative potential of neutrophils in RR-EAE mice was attenuated by AFN, likely reducing neuroinflammation and EAE-associated disease symptoms.

The induction of Nrf2 signaling not only activated antioxidant protective mechanisms but also reduced inflammatory and oxidative signaling related to the NF*k*B pathway [50]. NF*k*B is a master transcription factor required for the induction of inflammatory/oxidative proteins such as iNOS. The levels of oxidative mediators in the brain were reduced by AFN treatment through the downregulation of iNOS, MPO, and lipid peroxides in EAE mice, which could be due to the induction of Nrf2 signaling in the CNS. MPO is located in CNS plaques of subjects with MS, which could be associated with the infiltration of other leukocytes into the CNS in EAE mice [10,32,34,46,47,48,51]. The IL-6 levels were also increased in the CNS of EAE mice. The CNS also possesses resident immune cells such as microglial cells and infiltrated immune cells of myeloid/lymphoid origin, which have the potential to release inflammatory mediators such as IL-6 that cause neuroinflammation in the EAE model [34,49,52]. The AFN treatment caused a decrease in the levels of neuroinflammatory molecules and an attenuation of the clinical symptoms in the examined EAE model of RRMS, which could be due to its action ofnNrf2 signaling in multiple cells, including immune cells and neurons.

This study has some limitations. Firstly, the effects of a single dose of AFN were investigated, whereas a full log multiple-dose (at 0.5, 5, and 50 mg/kg) study would have provided better data. Secondly, apart from the cortex, other brain areas which may be affected by AFN and play an important role in the pathogenesis of EAE were not analyzed in this study. However, the cortex was chosen based on earlier studies, as the most prominent changes occur in this brain region after the onset of RRMS clinical symptoms, both in humans and in mice [53,54,55]. Thirdly, AFN may have direct effects on neuronal cells as well as immune cells in the CNS which can be better studied using an in vitro model system. Fourthly, a general marker for myeloid cells identification, i.e., CD11b, was used in this study; future studies should further examine specific myeloid cell markers such as Ly6G, F4/80, and CD11c to better understand the contribution of each myeloid cell. Apart from being expressed on myeloid cells, CD11b may also be expressed on non-myeloid immune cells such as NK cells. Lastly, apart from CD3+ T cells, other immune cells of lymphoid origin such as B cells, which may be critically involved in EAE pathogenesis, also need to be analyzed in future studies.

In conclusion, our study suggests that the AFN-mediated effects originated mainly from reduced TrxR activity and concurrent activation of Nrf2 signaling in the peripheral immune system and the CNS. The induction of Nrf2 signaling by AFN likely reduced the levels of oxidative and inflammatory mediators in the periphery and CNS in mice with EAE, thereby leading to the amelioration of the disease symptoms. Therefore, AFN might be a potential therapeutic molecule to reduce the neuronal/systemic inflammation associated with RRMS.

## Data Availability

All data presented in this study are available on reasonable request from the corresponding author.
